# High-Throughput Crystallization Pipeline at the Crystallography Core Facility of the Institut Pasteur

**DOI:** 10.3390/molecules24244451

**Published:** 2019-12-05

**Authors:** Patrick Weber, Cédric Pissis, Rafael Navaza, Ariel E. Mechaly, Frederick Saul, Pedro M. Alzari, Ahmed Haouz

**Affiliations:** 1 Plate-forme de cristallographie-C2RT, Institut Pasteur, CNRS UMR-3528, 75724 Paris, France; patrick.weber@pasteur.fr (P.W.); cedric.pissis@pasteur.fr (C.P.); rafael.navaza@pasteur.fr (R.N.); ariel.mechaly@pasteur.fr (A.E.M.); frederick.saul@pasteur.fr (F.S.); 2 Unité de Microbiologie Structurale, Institut Pasteur, CNRS UMR-3528, Université de Paris, 75724 Paris, France; pedro.alzari@pasteur.fr

**Keywords:** macromolecular crystallography pipeline, high-throughput crystallization screening, crystal optimization

## Abstract

The availability of whole-genome sequence data, made possible by significant advances in DNA sequencing technology, led to the emergence of structural genomics projects in the late 1990s. These projects not only significantly increased the number of 3D structures deposited in the Protein Data Bank in the last two decades, but also influenced present crystallographic strategies by introducing automation and high-throughput approaches in the structure-determination pipeline. Today, dedicated crystallization facilities, many of which are open to the general user community, routinely set up and track thousands of crystallization screening trials per day. Here, we review the current methods for high-throughput crystallization and procedures to obtain crystals suitable for X-ray diffraction studies, and we describe the crystallization pipeline implemented in the medium-scale crystallography platform at the Institut Pasteur (Paris) as an example.

## 1. Introduction

Recent decades have seen extraordinary advances in whole-genome sequencing techniques and bioinformatics annotations, but the challenge to assign a specific protein fold to each newly sequenced gene is yet to be achieved due to an ever-widening gap between genomic and structural information [1]. During the period of 2000–2015, a number of structural genomics (SG) centers (http://www.rcsb.org/stats/distribution_structural-genomics-centers) addressed this issue by implementing high-throughput methods using advanced technology for 3D-structure determination of biological macromolecules. These included semi-automated cloning of selected protein targets in prokaryotic and eukaryotic expression systems [2,3], robotic crystallization screening [4,5], and automated X-ray data collection at synchrotron beamlines [6]. SG programs generated enormous amounts of experimental data, resulting in the determination of over 13,500 protein structures [7]. Despite these numbers, SG programs have encountered criticism, mainly regarding funding matters, the target selection process, and the high attrition rates in the pipeline from genes to soluble proteins to diffracting crystals [8,9]. Although it may be too early to evaluate the long-term impacts of SG initiatives, the structural biology community and the scientific community at large have certainly benefitted from many technological advances developed by SG programs for biomolecular structure determination.

Many academic and industrial research institutions worldwide created core facilities with state-of-the-art equipment for high-throughput screening to obtain suitable crystals, which remains a major bottleneck for biomolecular crystallography. Some of these facilities offer open access to the general user community and have been in operation for many years (see, for example, [10,11,12,13]).

Despite the large number of crystal structures solved (around 10000 structures per year are deposited into the Protein Data Bank), the crystallization of biological macromolecules largely remains an empirical process. Briefly, to arrive at a supersaturation state necessary to induce crystallization, a protein of interest is mixed with organic or inorganic precipitants at different pH values at a constant temperature to vary the solubility of the sample [14,15,16,17]. Supersaturation can be reached within a few hours, days, months, or not all, depending on the protein under study and the method of crystallization (vapour diffusion, microbatch, microdialysis, or free-interface diffusion). These methods have quite different kinetics of crystallization, varying from a few hours to several months. While most of these methods have been routinely used for over 60 years, recent developments aimed at obtaining suitable crystals more rapidly with minimal investment of human resources and cost. In this respect, two major developments had a strong impact on the field: (i) Sparse matrix sampling methods [18] empirically derived from known or published crystallization conditions of various proteins, which led to the commercialization of the first crystallization screen kits, and (ii) the emergence of robotic technologies for miniaturization, automation, and high-throughput methods in the crystallization pipeline.

**Figure 1:**
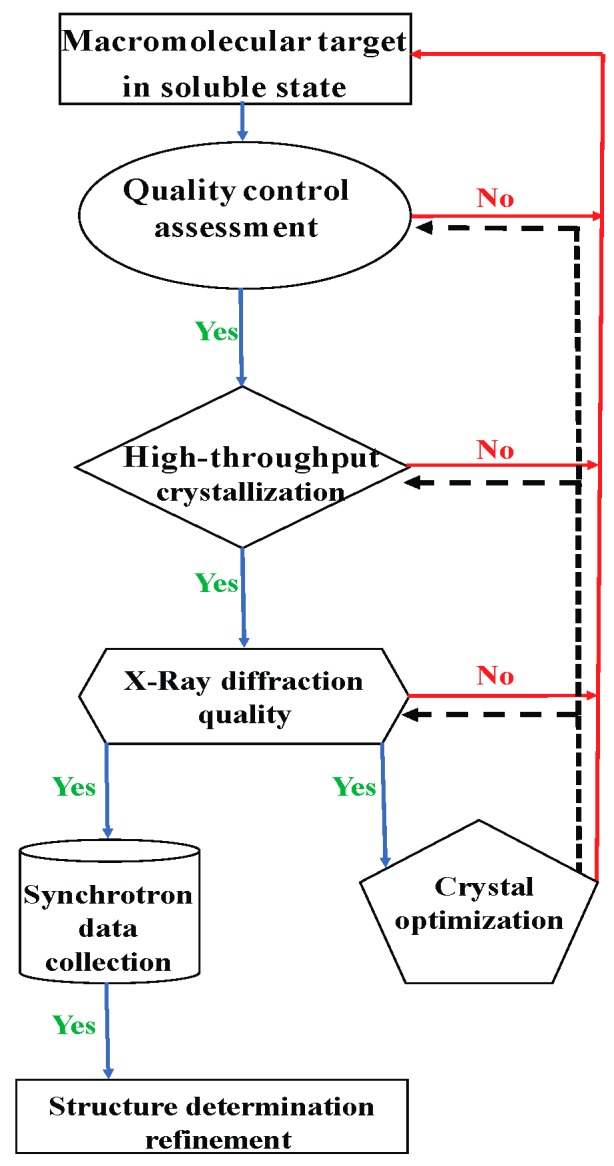
The macromolecular X-Ray crystallography pipeline.

The crystallization workflow in the macromolecular crystallography pipeline can be subdivided into three major steps (Figure 1). The first step is the pre-filling of crystallization plates (e.g., SBS standard 96-well plates) with the crystallization solutions. The second step is the generation of nanodrops containing a mixture of the protein sample with the crystallization solutions, and imaging of the drops to monitor crystal growth. The third step is devoted to the optimization of selected screening hits to obtain diffraction-quality crystals suitable for X-ray structural studies. This linear workflow, although repetitive and highly time-consuming, is particularly appropriate for the introduction of automation to reduce the need for manual intervention [19].

The latest advances in high-throughput protein crystallization screenings were recently reviewed [20]. In this article, we describe the experimental procedures and equipment available in the crystallography core facility at the Institut Pasteur, Paris, to allow users to obtain crystals suitable for X-ray crystallographic studies. Similar methodologies and equipment are available in many academic and industrial research facilities dedicated to molecular structural biology.

The workflow is shown step-by-step, starting with quality assessment of the purified soluble protein sample. For each step, success (Yes) is indicated by a green arrow, and failure (No) is indicated by a red arrow. At each step in the pipeline, a process of optimization (indicated by black dashed lines) can be carried out.

## 2. Results

### 2.1. Quality Control of the Protein Sample

For the most widely used techniques to determine macromolecular structures at the atomic level, namely X-ray crystallography, NMR spectroscopy and, increasingly, single-particle cryo-EM, ensuring the quality and integrity of the biological sample to be studied is a crucial prerequisite. To perform crystallization screening trials, the users of our core facility are strongly encouraged to perform a number of tests to control the purity, homogeneity, conformational integrity and, as far as possible, the biochemical function of the protein sample.

Many reviews have described in detail the biophysical and biochemical techniques that can be used for quality control of protein samples [21,22]. The protein purification process generally includes two or three steps, usually starting with affinity chromatography, followed by size exclusion and possibly ion exchange chromatography if the purity of the sample is not sufficient (less than 95%). Between successive purification steps, the user can monitor the purity and apparent molecular weight of the protein sample with sodium dodecyl sulfate polyacrylamide gel electrophoresis (SDS-PAGE) in denaturing conditions. Once the purity and integrity of the protein have been assessed, the sample must be concentrated to maximum solubility without aggregation. During this step, dynamic light scattering (DLS) [23] can be used to monitor the oligomeric state of the protein. This powerful, non-invasive technique reveals the presence of protein aggregates, poly- or mono-dispersity of the sample, and the apparent molecular weight of the protein under non-denaturing conditions. The primary sequence and the presence of post-translational modifications can be examined by mass spectrometry. The folding state of the protein can be monitored by NMR spectroscopy, circular dichroism (CD) at near- and ultra-UV, or Fourier-transformed infrared spectroscopy (FTIR). Finally, an evaluation of the biochemical function (enzymatic activity, ligand binding, etc.) is desirable to confirm the functional integrity of the protein sample.

It is best to perform these quality control procedures before conducting crystallization trials, rather than downstream in the pipeline after failing to obtain crystal hits. Most protein samples entering our crystallization pipeline are expected to be monodisperse (with polydispersity below 20% in DLS experiments), homogeneous (> 95% purity when assessed by SDS-PAGE), and at the highest possible concentration (not less than 5 mg/mL).

### 2.2. Management of Crystallization Kits

Since the introduction of the first crystallization screening kits in 1991 [18], more than 220 crystallization screens, allowing the exploration of ~13000 different conditions, are now commercially available [24]. These kits can be classified into three categories: (i) Sparse matrix screens containing an aqueous mixture of a few chemical compounds, often composed of salts, a biological buffer, and a polymer, (ii) grid screens in which the concentration of one precipitant agent is varied in a linear gradient versus pH, and (iii) additive screens typically containing 96 different small molecular weight compounds that can influence the crystallization process positively or negatively when added to the crystallization solution in small amounts. Some years ago, the C6 crystallography core facility analyzed the redundancy in the composition of all commercially available screening kits [24]. Using their website tools (http://c6.csiro.au/), we selected a low redundancy set of twenty commercials kits for our standard crystallization screening trials. These kits included sparse matrix, grid and additive screens that were particularly suitable for soluble or membrane proteins.

**Figure 2:**
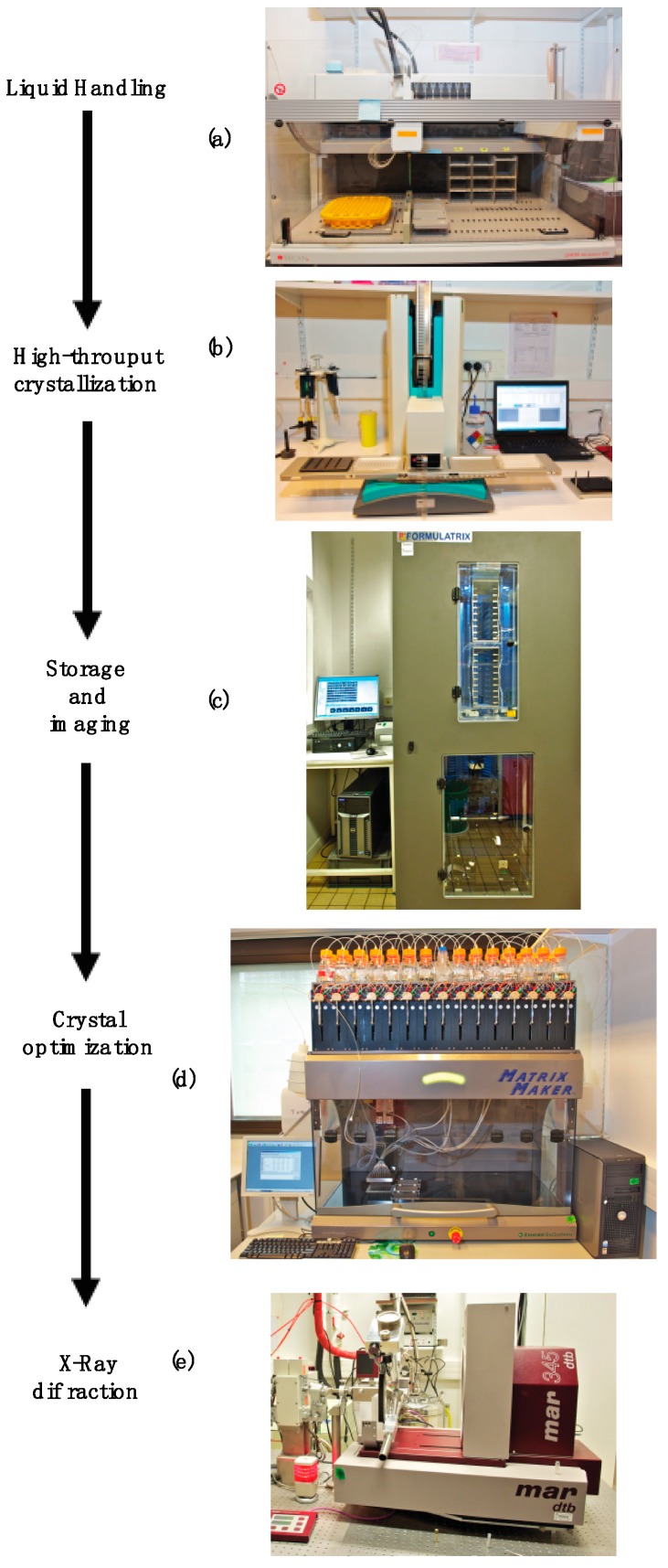
Equipment available in the X-ray crystallography platform. (**a**) TECAN Genesis 150 robot used to perform liquid handling experiments; (**b**) Mosquito robot (TTPlabtech) for nanodrop crystallization screening; (**c**) RockImager 1000 (Formulatrix) automated system for storage and imaging of the crystallization plates; (**d**) MatrixMaker automated system (Protein BioSolutions Inc.) to design solutions for crystal optimization; and (**e**) X-ray diffraction system to assess crystal quality.

To reduce costs, we usually purchase the screening kits in the 10 mL tube format and the solutions are transferred to 96-well deep well plates of 1.4 mL final volume using an automated TECAN liquid handling system (Figure 2). The plates are manually thermo-sealed with aluminum tape to avoid evaporation and stored at 4 °C. One of the deep-well blocks is reserved for users, allowing them to reproduce manually any crystal form obtained in robotic screens using the same solution. The remaining deep-well blocks are used to generate pre-filled crystallization plates with the liquid handling system by transferring 150 µL of the solution to each reservoir in the crystallization plate (Greiner bio-one, CrystalQuick, 96 well, Round well and U-bottom). The prefilled crystallization plates are manually sealed with aluminum tape and stored at 4 °C. For tracking purposes, each plate is labeled with the name of the commercial kit and the date of filling.

### 2.3. High-Throughput Crystallization Screening

The conditioning of the purified protein sample plays a crucial role in the reproducibility of crystallization experiments. The users of our facility are encouraged to contact us before the final purification steps of protein samples to be submitted for initial screening. Our strategy involves setting up automated crystallization trials with fresh protein samples that have not previously undergone any freezing/thawing process.

For archival and tracking purposes, the samples submitted for crystallization are accompanied by a request form with the following information:

1. User name, laboratory, project acronym, date of submission;
2. Sample name, concentration, volume, buffer, and temperature of storage;
3. Crystallization method: sitting/hanging drop vapour diffusion, cubic phase, or microbatch;
4. Crystallization drop: volume, ratio of protein/precipitant, volume of seed;
5. Temperature (4 °C or 18 °C);
6. Crystallization screens to be used, selected from a list of available kits.

This information is used to generate a barcode associated with each crystallization plate using RockMaker™ (Formulatrix) software. In order to minimize the overall cost of consumable items, up to three different protein samples can be simultaneously tested in each crystallization plate.

For microbatch and cubic phase screens, experiments are performed at 18 °C with a Douglas Oryx8 protein crystallization robot (Douglas Instruments Ltd., Berkshire, UK). For sitting or hanging drop vapor-diffusion experiments, screening trials at 4 °C or 18 °C are carried out by two independent Mosquito automated nanoliter dispensing systems (TTP Labtech, Melbourn, UK). The volume of the crystallization drop is typically 400 nL, with a 1:1 mixture of the protein sample and different screening solutions, but final drop volumes can range from 100 nL to several microliters. Typically, a drop of the protein sample is dispensed in the sub well of the crystallization plate before adding the drop of crystallization solution.

Each plate is manually sealed with transparent tape (CrystalClear HR4-506, Hampton Research, Aliso Viejo, CA, USA) to avoid evaporation and centrifuged at 1000 rpm for 10 min to ensure sufficient mixture of the protein sample and the crystallization solution before insertion into an automated imaging system. The number of crystallization plates set up for each project can vary depending on the amount of protein available and the specific goal of the screen, i.e., either identification of lead conditions by high-throughput screening or crystal optimization.

### 2.4. Storage and Imaging

In our facility, the crystallization plates are stored in a RockImager 1000 (Formulatrix, Bedford, MA, USA) automated storage and imaging system to monitor crystal growth. This system maintains the plates at constant temperature and allows automatic imaging of each crystallization drop according to scheduling determined by the facility (in agreement of the user). Periodic imaging of individual drops on each plate is performed to monitor the crystallization process. Each plate is routinely imaged at the following intervals: (1) Immediately after setting up the crystallization drops, (2) each day during the first week after setup, (3) every three days for the remaining time during the first month, (4) every week through the second month, (5) every two weeks through the third month, and (6) every month for the remaining time in the robot. The storage capacity of the RockImager system is 1000 plates and the average lifetime of each plate in the robot is around eight months.

The recorded images of each crystallization drop are transferred to a server database associated with the imaging system. Two RockImager robots are available in our facility, one set up at 18 °C and the other at 4 °C, which generate around ten million images per year. Users of the facility have internet access to images of the crystallization drops associated with their projects through a login account assigned to the hosting laboratory. The users are responsible for visual inspection and image scoring. The Rock Maker Web database management software, associated with the automated imaging system, is used as a laboratory information management system (LIMS) for the crystallization experiments; all relevant tracking information is contained in the plate barcode. The crystallization drops can be displayed along with details of the crystallization conditions and other relevant information to facilitate the scoring analysis of the results. With the RockMaker software, users can manually score the images of drops from 0 to 9: (0) clear drop, (1) dust, (2) granular precipitate, (3) full precipitate, (4) good precipitate, (5) phase separation, (6) microcrystalline, (7) needles, (8) plates, and (9) crystals. Manual inspection and scoring is a very time-consuming step and important efforts have therefore been dedicated to develop automated scoring methods based on machine-learning algorithms [25,26]. For instance, the recently described Machine Recognition of Crystallization Outcomes (MARCO) initiative (https://doi.org/10.4225/08/5a97375e6c0aa) is able to correctly categorize more than 94% of test images. This solution was implemented in the crystallization pipeline of the collaborative Crystallization Centre (C3) in Australia [27,28]. Automated image scoring based on MARCO was integrated in RockImager and RockMaker software for the Formulatrix imaging system (https://formulatrix.com/life-science-automation-blog/protein-crystallization-software-update-rock-maker-3-15/).

In our crystallization facility, the image scheduling procedure can be modified at any time based on the presence or absence of leads during manual scoring. If the initial screening trials fail to produce crystal hints, the user can explore other options depending on the protein sample. Thus, crystallization can be attempted in the presence of known protein ligands (coenzymes, substrates, inhibitors, etc.). If the target is a recombinant protein, new constructs involving point mutants or truncated versions of the protein can be screened, for instance, to eliminate purification tags or glycosylation sites that could interfere with crystallization, or to produce individual globular domains of a multi-domain protein. The crystallization of homologous proteins from other species can also be explored, especially if a thermo-stable equivalent is available. 

When single crystals of suitable size (> 5–10 µm) are obtained from the initial screening trials, they are directly harvested for X-ray diffraction analysis. For protein crystals that diffract to high resolution, a full dataset collected at this stage may be enough for structure determination (a situation encountered in around 20–30% of the projects submitted for crystallization screening in our facility). For poorly diffracting or very small crystals, the user must undertake a process of crystal optimization.

### 2.5. Crystal Optimization

Crystals are routinely cryo-cooled for data collection at synchrotron facilities to avoid radiation damage [29,30]. When large single crystals diffract poorly, different solutions can be tried to cryoprotect the crystal at liquid nitrogen temperature. The methods currently used to prevent crystal damage during flash-cooling were recently summarized [31]. In some cases, diffraction can be significantly improved by changing the nature or concentration of the cryoprotectant mixed with the crystallization solution.

If initial cryo-cooling experiments are unsuccessful, the crystallization conditions (concentration of precipitant, pH, divalent ions, additives, etc.) can be varied to improve crystal quality. These experiments are time-consuming and would normally require significant manual intervention. To overcome this bottleneck, we offer access to a MatrixMaker™ (Protein BioSolutions Inc) automated liquid handling system to generate a wide range of crystallization solutions. More than 150 different mother solutions are currently managed by the facility, and we standardized the fabrication procedure for the mother solutions in a database associated with the MatrixMaker™ system by assigning a unique name for each chemical compound. For tracking purposes, the compounds present in this database are referenced by manufacturer, catalog reference, chemical formula and CAS registry number. In order to follow the proposed crystallization ontology [32], users are strongly encouraged to adopt this nomenclature in publications and protein data bank (PDB) data deposition.

CryMon software (Protein BioSolutions, Gaithersburg, MD, USA), which is used to design a specific screening matrix, is installed in each user’s laboratory. The design of crystallization solutions can be monitored externally through a network server connected to the MatrixMaker system. Other automated systems to design customized screens, such as the Scorpion (Art Robbins), Dragonfly (TTPlabtech), and Formulator (Formulatix), are also commercially available. These systems differ mainly in the number of mother solutions managed simultaneously, and overall setup times (for illustration purposes, more than 800 customized screens formatted in 24-well crystallization plates, deep-well blocks, or 50 mL tubes are designed per year in our facility). The solutions are used to generate custom grid screens or to design additive matrices close to the crystallization condition to be optimized. The chemical compounds most frequently used to design these matrices are ammonium sulphate, low- and medium-molecular weight polyethylene glycol (PEG) and buffers with Hepes-HCl or Tris-HCl; these results are comparable to published statistics regarding the most popular chemical compounds used for the crystallization of biological macromolecules [33].

If the optimization procedure fails to improve diffraction quality, existing crystals (or promising crystalline material) can still be used to set up seeding experiments. Microseeding was shown to be an effective strategy for recalcitrant crystallization projects [34]. This method can be implemented robotically for automated screening by dispensing an aliquot containing crystal seeds into crystallization drops unrelated to the seed source. Another possibility is to modify the packing of protein molecules in the crystal by testing the effect of the presence or absence of a fusion tag used to improve the yield and solubility of the recombinant protein, or by adding an aliquot of protease to the protein sample before setting up the crystallization drop to eliminate possible disordered regions of the protein that might influence intermolecular contacts in the crystal lattice [35,36]. Finally, crystallization of a homologous protein from another species can be attempted. As an example, after unsuccessful attempts to crystallize close homologs of malotose transporter (MalT) STAND-ATPase from different proteobacteria, we were able to produce high-resolution diffracting crystals of *Pyrococcus horikoshii* PH0952, a related MalT homolog from archaea [37].

### 2.6. X-Ray Diffraction and Data Collection

Our facility includes an in-house X-ray diffraction system, including a Rigaku MicroMax-007 microfocus rotating anode generator, a MarResearch 345dtb image plate detector, and an Oxford 600 cryo-cooling system. Historically, this system was a useful element in the crystallography pipeline to (i) assess the diffraction quality of crystals obtained from the initial screens and determine if they are from the protein sample or small molecules (salt) present in the crystallization solution, (ii) define suitable cryoprotectant solutions for crystal cooling in liquid nitrogen, and (iii) screen crystals for the best diffraction quality (resolution, spot shape, mosaicity, etc.) for subsequent X-ray data collection at synchrotron facilities. 

Nowadays, the usefulness of maintaining an in-house X-ray diffraction system for macromolecular crystallography is a matter of debate, particularly for those teams with convenient access to synchrotron facilities. Indeed, the technological advances brought into the field by structural genomics projects have also greatly influenced automation in X-ray data collection at synchrotron beamlines. Cryo-cooled crystals are mounted and centered on the goniometer by a robotic arm [38] and diffraction data can be automatically collected at different wavelengths [39]. For high-throughput data collection, brighter synchrotron beams coupled to faster X-ray detectors, such as the Dectris pixel-array PILATUS [40] or EIGER [41] detectors, reduce the time needed to collect a full dataset to just a few minutes, or seconds. Today, crystallographers can collect full X-ray datasets from over 20 crystals in one hour, compared to only four crystals per hour in 2007 [6]. At some synchrotron beamlines, fully automatic crystal characterization and data collection are now routinely used by academic and industrial research groups with minimal human intervention [42]. For more details, a recent review nicely summarized the technological developments at synchrotron facilities ranging from detectors to optics and automation [6]. 

Finally, computing plays a central role in the resolution of macromolecular structures. Our facility provides users with computing infrastructure and crystallographic software for X-ray data processing, phase determination, model building, refinement, and structure validation. To ensure this service, a full-time systems manager is necessary to administer and maintain user workstations (currently more than 100 specially configured Mac and Linux workstations at Institut Pasteur) and servers (Solaris, FreeBSD, Linux, and Windows). The role of the systems manager also includes the installation and updating of crystallographic software packages and ensuring backup/storage of synchrotron X-ray diffraction data. As of October 2019, our current data storage capacity is 100 Tb. 

## 3. Conclusions and Future Prospects

X-ray crystallography is the most widely used technique to reveal the three-dimensional structure of biological macromolecules at atomic resolution. These structures provide information on the biological function, reaction mechanisms, and interactions of a protein with substrates or effectors. Access to high-throughput crystallization facilities equipped with state of the art equipment has greatly benefitted the community for structural studies; a website describing the equipment available and access modalities for a number of these facilities is accessible at https://research.csiro.au/crystal/references/other-crystallisation-centres/. For example, the C3 collaborative crystallization center in 2019 set up a total of 1,724 crystallization plates for 66 users, and the HTX Lab at EMBL-Grenoble has performed, to date, more than 10 million crystallization experiments for 900 users from 23 different countries. Our crystallography facility is capable of processing more than 1000 protein samples per year, provided by more than 80 users. The biological macromolecules studied at Institut Pasteur are of major interest in the field of life sciences related to human health, and the goal of our crystallographic facility is to assist users to efficiently solve the 3D structures of biological macromolecules and their complexes for drug discovery, diagnostic applications, and vaccine development.

For several decades, X-ray crystallography has been a formidable engine to study molecular biology at the atomic level and, judging by the number of structures deposited each year in the Protein Data Bank, remains the primary technique to determine the 3D structure of biological macromolecules. Today, biocrystallography faces new opportunities and challenges with the extraordinary advances in single-particle cryo-electron microscopy [43]. Addressing challenging structural biology projects with complementary techniques such as X-ray crystallography and cryo-EM (single-particle analysis and tomography) will allow us to study heretofore intractable macromolecular assemblies to shed light on the cellular mechanisms that govern physiological and physiopathological processes.
